# Retraction: Proline-Rich Tyrosine Kinase 2 (Pyk2) Regulates IGF-I-Induced Cell Motility and Invasion of Urothelial Carcinoma Cells

**DOI:** 10.1371/journal.pone.0215889

**Published:** 2019-04-18

**Authors:** The PLOS ONE Editors

Following publication of this article [1], the following concerns were raised:

- Fig 4B Pyk2 panel lanes 1–2 appear similar and contain vertical discontinuities between them.
- Grb2 panel lanes 3–4 appear similar in Fig 4B and 4C.
- Fig 4C Pyk2 panel lanes 3–4 appear similar.
- Fig 4C Grb2 panel lanes 1–2 appear to have background discontinuities above and below the bands.

The authors have commented that they do not agree with the concerns and stand by the integrity of their data and the conclusions of their publication. The authors provided further images in support but stated that owing to the time that has passed, the uncropped scans of the films are no longer available and thus were unable to resolve the above concerns. In the absence of the data underlying the published Fig 4B,C and in light of the concerns affecting these panels, the *PLOS ONE* Editors retract this article.

MG, SQX, LGG, AB, RVI, and AM did not agree with retraction. SB and SCP did not respond.
